# The Hidden Side of NCAM Family: NCAM2, a Key Cytoskeleton Organization Molecule Regulating Multiple Neural Functions

**DOI:** 10.3390/ijms221810021

**Published:** 2021-09-16

**Authors:** Antoni Parcerisas, Alba Ortega-Gascó, Lluís Pujadas, Eduardo Soriano

**Affiliations:** 1 Department of Cell Biology, Physiology and Immunology, Institute of Neurosciences, University of Barcelona, 08028 Barcelona, Spain; albaortega@ub.edu (A.O.-G.); lluis.pujadas@ub.edu (L.P.); 2 Centro de Investigación Biomédica en Red Sobre Enfermedades Neurodegenerativas (CIBERNED), 28031 Madrid, Spain; 3 Department of Basic Sciences, Universitat Internacional de Catalunya, 08195 Sant Cugat del Vallès, Spain

**Keywords:** NCAM2, cytoskeleton, Actin, microtubule, MAP2, CaMKII, Autism Spectrum Disorder, Alzheimer’s disease

## Abstract

Although it has been over 20 years since Neural Cell Adhesion Molecule 2 (NCAM2) was identified as the second member of the NCAM family with a high expression in the nervous system, the knowledge of NCAM2 is still eclipsed by NCAM1. The first studies with NCAM2 focused on the olfactory bulb, where this protein has a key role in axonal projection and axonal/dendritic compartmentalization. In contrast to NCAM1, NCAM2’s functions and partners in the brain during development and adulthood have remained largely unknown until not long ago. Recent studies have revealed the importance of NCAM2 in nervous system development. NCAM2 governs neuronal morphogenesis and axodendritic architecture, and controls important neuron-specific processes such as neuronal differentiation, synaptogenesis and memory formation. In the adult brain, NCAM2 is highly expressed in dendritic spines, and it regulates synaptic plasticity and learning processes. NCAM2’s functions are related to its ability to adapt to the external inputs of the cell and to modify the cytoskeleton accordingly. Different studies show that NCAM2 interacts with proteins involved in cytoskeleton stability and proteins that regulate calcium influx, which could also modify the cytoskeleton. In this review, we examine the evidence that points to NCAM2 as a crucial cytoskeleton regulation protein during brain development and adulthood. This key function of NCAM2 may offer promising new therapeutic approaches for the treatment of neurodevelopmental diseases and neurodegenerative disorders.

## 1. Introduction

Neurons express a large variety of membrane proteins involved in multiple processes. These proteins act as a link between the extracellular environment and the intracellular compartment, and mediate different processes. Cell adhesion molecules (CAMs) include more than 200 proteins that are classified into four families [1]: cadherins, immunoglobulin superfamily (IgSF), integrins and neurexins/neuroligins. CAMs display a large structural variety, which reflects the functional diversity of these proteins. Typically, CAMs are involved in cell signaling [2,3], cytoskeleton remodeling [4] and the regulation of gene expression [5]. Several studies and hypotheses relate particular combinations of CAMs to a specific molecular identity of each neuron. This identity can be crucial for the processes that take place during brain development and adult maintenance, such as axon guidance, membrane recognition and neuronal network formation [6,7,8,9,10,11]. In Drosophila this molecular identity is conferred by *dscam*, which has multiple splicing sites that produce different isoforms of Down Syndrome Cell Adhesion Molecule, DSCAM. The particular pattern of isoforms expressed in neurons allows a correct membrane recognition and neuronal network formation [11,12]. Flies without *dscam* present multiple dysfunctions in axon guidance and dendritic tree development [13]. In mammals, according to the current hypotheses, neuronal molecular identity is determined by the expression of different CAMs. Although mammalian CAMs have parallel functions and their distribution is similar, they confer a specific molecular identity and are involved in several processes during brain development and synaptic plasticity, such as the proper formation of visual and hippocampal circuits [6,7].

Several functions of CAMs are correlated with their ability to transduce the external inputs to the cytoskeleton structure. During brain development, a proper regulation of cytoskeleton stability and dynamics are crucial for neuronal migration, cell differentiation and synapse formation [14]. In the adult brain, the cytoskeleton participates in synaptic maintenance and plasticity, and regulates the composition of presynaptic and postsynaptic compartments. There are various genomic studies that link adhesion proteins with mental retardation and psychiatric disorders, such as autism and schizophrenia [15,16,17]. These pathologies show altered neuronal development and connectivity [18]. The different functions performed by CAMs make them target molecules of interest in these pathologies [7], Table 1.

Among CAMs, the immunoglobulin superfamily (IgSF) represents one of the oldest and most diverse families. IgSFs have an extracellular part with different immunoglobulin and fibronectin domains, a transmembrane domain and a cytosolic tail through which they interact with the cytosol. Different families exist in the IgSF superfamily: L1 Cell Adhesion Molecule (L1CAM); Contactins; DSCAM and Neural Cell Adhesion Molecule (NCAM).

**Figure 1:**
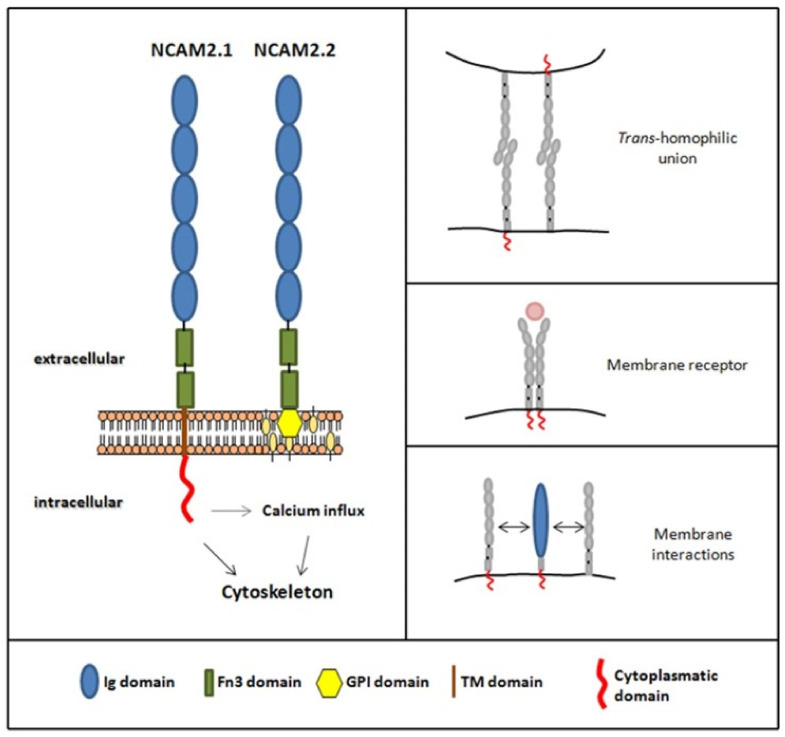
Schematic representation of NCAM2 protein and isoforms. Both isoforms have an extracellular part with five immunoglobulin domains (blue) and two fibronectin type III domains (green). NCAM2.1 isoform bears a transmembrane domain (brown) and a cytoplasmic domain (red), whereas NCAM2.2 isoform is glycosylphosphatidylinositol, GPI, anchored (yellow). It has been described that NCAM2 protein could do different type of interactions, such as trans-homophilic unions, membrane interactions and could also act as a membrane receptor.

The mammalian NCAM family has two members: NCAM1 and NCAM2 [53,54]. The two proteins have similar ectodomains, each of them containing five immunoglobulin domains and two fibronectin type III domains [55], Figure 1. NCAM1 was the first cell adhesion molecule identified in the nervous system and has been extensively studied. The different isoforms of NCAM1 (180KDa, 140KDa and 120KDa) have the same extracellular domain, but different transmembrane and cytosolic domains as a result of alternative splicing of the gene. The NCAM180 isoform is mostly expressed in neurons in the adult brain; NCAM140, in neurons in both embryonic and adult stages; and NCAM120, mostly in glia in adult stages.

NCAM1 participates in multiple processes in the brain. NCAM1 controls neurite growth, axonal and dendritic elongation, neuronal migration and cell positioning. Those functions are regulated through homophilic and heterophilic interactions of NCAM1 that activate different pathways ranging from calcium signaling to cytoskeleton modifications or transcription activation. Some of the functions undergone by NCAM1 are dependent on the polysialylation of NCAM1 and the ability of NCAM1 to interact with the cytoskeleton [56]. The polysialylation levels of NCAM1 increase during embryonic stages to progressively decline in postnatal stages [57] and it has been described that mouse models deficient in the enzymes responsible for polysialylation present an altered expression of transcription factors and show defects in migration. The functions of this cell adhesion molecule are also relevant in the processes of synaptogenesis and synapse maintenance. NCAM1 is one of the first molecules to accumulate at forming synapsis, promoting membrane binding between neurons and the stabilization of the connection. Indeed, the overexpression of the protein led to a stimulation of the formation of synapses while a deficiency results in a reduction in the total volume of dendritic spines [58,59,60]. NCAM1 is important not only for synapse formation but also for the regulation of synapse dynamics due to its interaction with different cytosolic (e.g., actin and microtubules cytoskeleton) and membrane components from the presynaptic and postsynaptic compartments [56,57,58,59,60,61,62]. Overall, NCAM1 is a key protein in neuronal network formation in the excitatory system but also in the inhibitory connections as it is important for the maturation of GABAergic synapses [63,64]. Moreover, modifications in NCAM1 protein levels due to genetic or environmental factors are associated with different neuronal pathologies [61,62].

In contrast to the wealth of knowledge currently available for NCAM1, NCAM2 has been less extensively studied and therefore its functions are still poorly understood, relegating this member of the protein family to a dark area for many years. However, over the last few years, a number of studies have revealed the importance of NCAM2 in neuronal morphogenesis, synapse formation and synaptic plasticity, with some of these functions being related to calcium dynamics and the ability of NCAM2 to modulate the cytoskeleton. In this review, we aim to shed light on the dark side of the NCAM family by focusing on the function of NCAM2 as a key molecule in the organization of the cytoskeleton.

## 2. NCAM2 Expression and Interactors

The NCAM2 gene is believed to have been originated by genetic duplication of *ncam1*, as they are paralog genes [53,54]. In humans, the NCAM2 gene is located on chromosome 21 (region 21q21.1) and contains 25 exons. In mice, it is located on chromosome 16 (16 C3.3) and contains 19 exons. In both species, and due to an alternative splicing process, two isoforms are transcribed: *ncam2.1* and *ncam2.2*. Compared to the mRNA of the *ncam2.1* isoform, which contains a stability element localized to the 3’-UTR region [65], the mRNA of the *ncam2.2* isoform is more unstable, there is less expression and it is more temporarily restricted [66]. In addition to the isoform differences at the transcript level, the two resulting proteins are also different: the NCAM2.1 isoform bears transmembrane and cytoplasmic domains, whereas the shorter NCAM2.2 isoform is glycosylphosphatidylinositol, GPI, anchored [65,66], Figure 1.

NCAM2 is mostly expressed in numerous central nervous system areas, including the cerebral cortex, the hippocampus and the olfactory system. Several studies showed expression of the murine NCAM2 gene in cortical areas, from embryonic day (E) 14 onwards, with the protein localizing to both dendritic and fiber compartments [65,67]. At adult stages, there is a switch in NCAM2 cell localization that results in higher levels of the protein being present in synaptic contacts [68,69].

The NCAM2 protein undergoes different post-translational modifications including palmitoylation, phosphorylation, proteolysis and glycosylation. Firstly, the cytosolic domain of NCAM2.1 contains four cysteine residues that may undergo palmitoylation: C723, C729, C734 and C740 [70]. Palmitoylation is an important modification for proteins to cluster into lipid raft domains [4,71,72]. Secondly, three phosphorylated residues in the cytoplasmatic tail have been described in murine samples from developing brains: S765, T780, and S786 [73] and the cytosolic tail of NCAM2 is a PEST region—that is, a peptide sequence that is rich in phosphorylatable residues: proline (P), glutamic acid (E), serine (S), and threonine (T) [74]. Besides, the cytosolic tail of NCAM2.1 presents SH2 and SH3 domains. Many proteins with these domains are involved in cell signaling through the activation of tyrosine kinases, such us Tyrosine-protein kinase Fyn or focal adhesion kinase, FAK. Thirdly, both NCAM2 isoforms present a proteolytic site between amino acids 682 and 701; proteolysis at this site causes the extracellular fragment to be released [75,76]. Fourthly, the extracellular region has eight N-glycosylation sites which are HNK-1 sequences. It has been reported that HNK-1 sequences and glycosylation present in L1CAM and myelin-associated glycoprotein, MAG, could facilitate migration of neural-crest-derived cells [77]. Noteworthy, and despite the high structural similarity of NCAM2 and NCAM1, polysialylation modifications have not been reported in NCAM2 proteins.

Few studies have addressed to NCAM2 interactions; these are summarized in Table 2. NCAM2 establishes homophilic bonds through its first immunoglobulin domain. Such bonds facilitate interactions in *cis*, which lead to dimerization of the protein, and in *trans*, which facilitate binding between different neurons [78]. Various studies have described interactions of the extracellular part of NCAM2 with prion protein (Prp), beta-amyloid peptide, fibroblast growth factor receptor (FGFR), epidermal growth factor (EGFR), Beta-site APP cleaving enzyme 1 (BACE1), Nogo and granulin (GRN) [70,78,79,80,81,82]. Other studies have shown interactions of the cytosolic tail of NCAM2.1 with members of the 14-3-3 protein family; Proto-oncogene tyrosine-protein kinase, c-Src; microtubule associated proteins, MAPs; neurofilaments; NFs, Calcium/calmodulin-dependent protein kinase type II, CaMKII; and F-actin-capping protein, CAPZ [69,70,76,78,81,82,83], Figure 1.

## 3. NCAM2 in Neuronal Cell Fate Determination and Differentiation

During neuronal development, the differentiation of a neuron from a postmitotic cell encompasses different processes [84]. Imbalances in the homeostasis of the cytoskeleton dynamics of the postmitotic cell give rise to neuronal polarization, which results in the formation of dendrites and an axonal terminal [85,86,87]. This polarization process is intrinsic to the differentiating neuron but modulated through external signals. Different CAMs are involved in neuronal polarization, act as transducers with the outside, and are key in the overall neuronal development and differentiation processes both in vivo and in vitro [14,88]. Studies with NCAM2 revealed that this protein is essential in the process of neuronal differentiation [69,83]. The distribution of NCAM2 inside the neuron and its interactions with proteins that regulate the cytoskeleton dynamics are key to NCAM2 participating in the establishment of both the dendritic tree and the axonal process.

### 3.1. NCAM2 Role in Neuronal Migration and Corticogenesis

**Figure 2:**
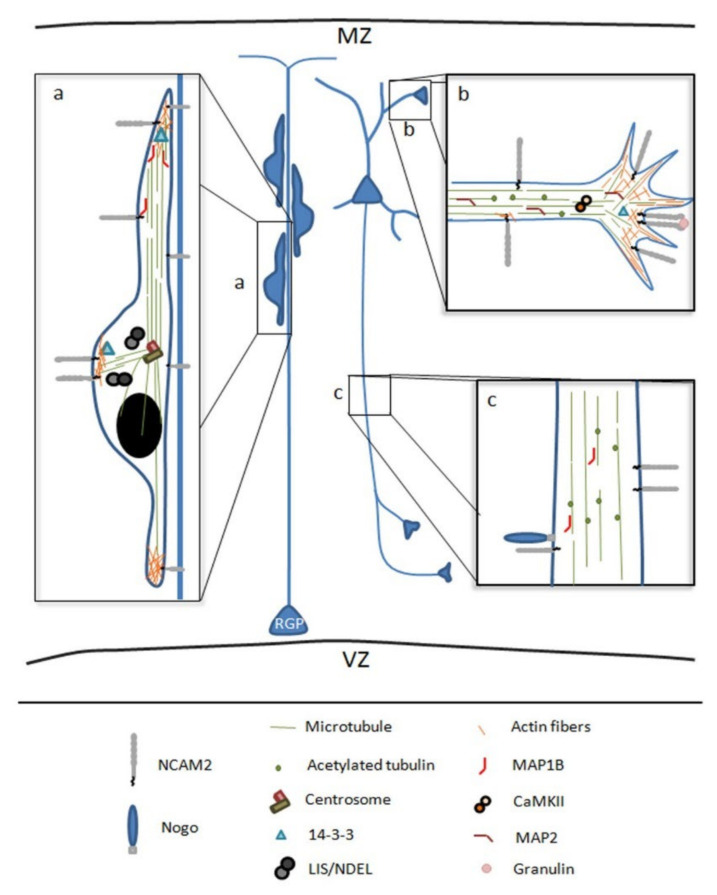
Schematic representation of NCAM2 functions and interactions during brain development. (**a**) NCAM2 is involved in neuronal migration, NCAM2 protein levels have been associated with an aberrant neuronal migration and layering positioning in the cortex. NCAM2 interacts with different cytoskeletal associated proteins which control cytoskeleton dynamics during neuronal migration. (**b**,**c**) NCAM2 has a crucial role during neuronal differentiation interacting with different proteins that are necessary for dendritic tree development and axon elongation. NCAM2 depletion alters the growth cone mobility and microtubules stability. Microtubule associated protein 1B, MAP1B; Microtubule associated protein 2, MAP2; and Calcium/calmodulin-dependent protein kinase type II, CaMKII. Lissencephaly-1/Nuclear distribution protein nudE-like1, LIS/NDEL; radial glia progenitors, RGP; marginal zone, MZ; and ventricular zone, VZ.

Brain formation involves a myriad of mechanisms, including temporal and spatial regulations during neuronal development that only occur in vivo [14]. The study of the mechanisms that regulate the polarization and development of neurons in vitro made it possible to identify proteins that have an implication at the physiological level [89]. Not only that, but the complexity of in vivo models has demonstrated the relevance of the extracellular matrix and cell adhesion molecules in the overall development of the brain. In this sense, adhesion proteins participate in different processes of neuronal development, such as neuronal migration, positioning, morphogenesis, development of dendritic and axonal compartments, and synaptogenesis [58,88,90,91]. In vivo, NCAM2 expression progressively increases during neuronal development; NCAM2 is involved in neuronal migration, cell positioning during corticogenesis, neuronal differentiation and synaptogenesis in the olfactory bulb, neocortex and hippocampus. In these regions, NCAM2 participates in the development of dendrites and axons, the establishment of connections, and the regulation of synaptic plasticity [66,68,92], see Figure 2.

In mice embryonic stages, NCAM2 is expressed in different brain regions. In the neocortex, NCAM2 expression is detected from the area of neuronal neurogenesis, ventricular zone and subventricular zone, to the cortical plate. In the olfactory bulb, NCAM2 has been shown to be important for establishing synaptic connections in the glomerulus. In animals constitutively deficient in NCAM2, defects have been described in the formation of olfactory bulb connections, leading to abnormal electrophysiological patterns [93,94]. However, no alterations have been reported in the formation of cortex in animals constitutively deficient in NCAM2 [94]. In contrast, using an in utero electroporation approach, changes in NCAM2 protein levels have been associated with an aberrant neuronal migration and layering positioning in cortex [69] (Ortega-Gascó 2021, data unpublished). The discrepancy between results obtained in mice constitutively deficient in NCAM2 and mice where NCAM2 was manipulated through in utero electroporation could be explained because in utero electroporation allows researchers to cause changes in NCAM2 levels sharply and specifically, thus avoiding any possible compensatory effects.

Neuronal migration and brain layering are crucial processes for the formation a well-structured brain. During neuronal migration, a proper regulation and functioning of cytoskeleton dynamics is involved in the leading process, the nucleokinesis and the tailing process [95]. The downregulation of NCAM2 in vivo causes an alteration of cortical migration, which in turn leads to a mislocalization of layer II-III fated neurons and an altered morphology [69]. NCAM2 overexpression results in a delay in neuronal migration during cortical development (Ortega-Gascó 2021, data unpublished). The molecular mechanism through which NCAM2 is affecting migration is unknown, but there are indications that it could involve radial migration or terminal somal translocation. NCAM2 interacts with several proteins that are involved in the regulation of migration through the Lissencephaly-1/Nuclear distribution protein nudE-like1 (LIS/NDEL) complex, such as 14-3-3ε or the light chains of the dyneins, Dyll1 and Dyll2. The LIS1/NDEL protein complex is key to the process of nucleokinesis in radial migration through the control of microtubule dynamics and centrosome positioning [96,97,98,99]. Heterogeneous deletions of the LIS1 gene have been observed to induce lissencephaly and Miller–Dieker syndrome. In these pathologies, defects in neuronal migration occur that prevent the correct positioning of neurons and the formation of folds in the cortex [100]. The LIS/NDEL complex interacts with different proteins that control its positioning and activity: dyneins, cell division kinesin 5, Aurora A and 14-3-3ε [101,102,103,104]. The interaction of the complex with cytoplasmic dyneins is key to the positioning of the complex during nucleokinesis. The interaction of 14-3-3ε with the complex is essential for the maintenance of NDEL phosphorylation, which controls the complex activity [103]. Thus, 14-3-3ε-deficient animals have erroneous neuronal migration, leading to aberrant brain development that resembles the phenotype of the LIS1-deficient animal [96,103,105]. NCAM2 interacts with MAP1B, which also participates in the regulation of neuronal migration. Changes in MAP1B protein levels disrupt the migration process. In particular, MAP1B controls microtubule stability and reorientation [95,106]. So, NCAM2 interacts with proteins involved in the positioning and functioning of the LIS/NDEL complex and in the correct arrangement of the microtubule cytoskeleton. The results obtained about NCAM2.1 and NCAM2.2 overexpression point to their role in transition from multipolar to bipolar fate due to overactivation of intracellular signaling, resulting in cell retention in the subventricular and intermediate zones (unpublished data). During the transition from multipolar to bipolar fate, there is a retraction of neurites and a rearrangement of the cytoskeleton [88]. The increase of NCAM2 levels produces more protein interaction in the membrane domain (Ortega-Gascó 2021, unpublished data). In the same way, TAG-1 is also an extracellular cell adhesion molecule anchored to the membrane with a GPI domain, and its deficiency in neurons prevents the transition from multipolar to bipolar and the axon specification [107,108]. The mechanism by which TAG-1 produces this phenotype is based on *cis* interactions with Src family proteins present in the raft lipid domains [107,108].

### 3.2. Neuronal Differentiation

NCAM2 is expressed in dendritic and axonal compartments and several studies showed its crucial role in neurite outgrowth, dendrite development and axon elongation.

#### 3.2.1. NCAM2 in Dendritic Tree Development

The activation of NCAM2 produces an increase in the number of filopodia and the length of neurites due to an increase in the intracellular calcium levels, which activate the CaMKII complex [83]. Conversely, the knockdown of *ncam2* expression during dendrites’ formation causes the retraction of existing neurites and the appearance of new processes from the soma [69]. These phenomena produce a significant increase in the number of primary dendrites; however, these dendrites are shorter, have more branching points, and (at a qualitative level) they are thinner compared to the control situation. 

The above dendritic tree alterations could be explained by the interaction of NCAM2 with MAP2. Different studies showed interaction and colocalization of NCAM2 and MAP2 in in vitro neuronal cultures and cingulate cortex [68,69,92]. MAP2, a microtubule-binding protein that promotes microtubule formation and stabilization [109], is key to dendritic formation [86]. In in vitro cultures, a decrease in MAP2 levels impedes the formation and differentiation of dendrites [110]. Not only that, but MAP2-deficient mice exhibit alterations and a reduction in dendritic length [111,112]. Several researchers have hypothesized that NCAM2 could contribute to dendritic tree development and corticogenesis in mouse models. The NCAM2-MAP2 interaction reveals the mechanism through which a loss of NCAM2 produces a reduction in MAP2 levels in dendrites, which in turn alters dendrite formation [69]. In line with this, a microtubule stabilization drug such as taxol reverts the effects of NCAM2 depletion in MAP2 protein levels and dendritic tree formation [69].

Aside from interacting with MAP2, NCAM2 interacts with the proteins of the CaMKII and 14-3-3 families [69,83]. CaMKIIα and CaMKIIβ, two subunits of the CaMKII complex, are involved in neurite formation and dendritic tree development. The CaMKII complex activates different signaling pathways that cause cytoskeletal modifications and activate transcription factors necessary for neuronal differentiation [113,114]. It has been shown that activation of CaMKII through different adhesion molecules, such as NCAM2, is required for neuronal differentiation [83,115]. The 14-3-3 proteins regulate the dynamics of the actin cytoskeleton and the microtubules [116,117]. In particular, 14-3-3ζ controls neurite growth through Protein kinase A, PKA [118] or GSK3β activation [119]. 

MAP2 transcription is controlled by 14-3-3ζ and L1CAM. 14-3-3ζ controls MAP2 transcription via the PI3K/Akt/NF-κB signaling pathway [120], while L1CAM controls the transcription of MAP2 via Src and the MAPK signaling pathway [121]. The effect of NCAM2 activation on gene transcription is still unknown, although NCAM2 activates Src [83]. NCAM2 interactions and calcium flux could activate different transcription factors that enhance the expression of proteins involved in neuronal morphogenesis, such as MAP2, thus producing a loop that would facilitate dendritic development.

#### 3.2.2. NCAM2 in Axon Formation and Development

Different mechanisms are involved in the branching and elongation of the axonal terminal, some of which required a certain level of cytoskeleton stability [122]. In this sense, excessive instability of the microtubule cytoskeleton results in a higher number of branching points [123]. NCAM2 deficiency increases the number of branches and reduces the maximum length of the main axon, without altering the total length of the axonal shaft [69]. Moreover, 20% of NCAM2-depleted neurons exhibit two or more axons. At the cytoskeleton level, NCAM2 deficiency reduces the signal of acetylated tubulin in axons. Tubulin acetylation is a posttranslational modification that occurs only when microtubules are polymerized and thus is an indirect measure of microtubule cytoskeleton stability [124]. Regarding axon elongation, NCAM2 interacts with MAP1B, which binds to the microtubules and regulates their dynamics. The regulation of MAP1B binding to microtubules is crucial during the axon branching process and changes according to the degree of phosphorylation [125]. In addition, NCAM2 interactions with the light- and medium subunits of neurofilaments have been identified. During development and neuritogenesis, these two subunits form heterodimers and participate in the cytoskeleton dynamics required for axonal growth and transport [126]. Through direct interaction with these neurofilament subunits, with MAP1B, or through signaling pathways that modulate said stability, NCAM2 has a role in stabilizing microtubule cytoskeleton to ensure a proper axon development in terms of length and branching points.

A key structure during neuronal differentiation is the growth cone. Growth cones are located at the end of both dendrites and axons, and explore the extracellular environment [127]. NCAM2 has been shown to modify the dynamics of the growth cone: a NCAM2 deficiency causes the protrusions coming out of the soma to be more dynamic and to present an aberrant morphology, as compared with the control situation [69]. Growth cone dynamics require a proper structure of the actin and microtubules cytoskeletons [127]. The fact that NCAM2 interacts with actin and the CAPZ complex (CAPZ2α and CAPZβ) is relevant since these proteins are involved in growth cone morphology and function. Specifically, the loss of a CAPZ subunit produces an aberrant growth cone with an ectopic position of the microtubules due to a failure in CAPZ binding capacity to tubulin [128]. At the same time, the NCAM2 interactors MAP2, MAP1B and proteins of the 14-3-3 family interact with the actin and microtubule cytoskeleton facilitating the organization and function of the growth cone [118,129].

In mature neurons, as opposed to other cell types, most microtubules do not depend on the centrosome and are polymerized by non-centrosomal mechanisms [130,131]. The mechanisms by which these phenomena occur are not known in detail; several mechanisms are believed to be involved. In relation to the polymerization process, it has been observed that a reduction in NCAM2 levels reduces the non-centrosomal polymerization in axons and dendrites. This finding, together with a reduction in acetylated tubulin levels upon a reduction in NCAM2 levels, indicates a greater presence of tubulin not bound to microtubules. So, when NCAM2 is depleted the equilibrium in the ratio of polymerized versus free tubulin is displaced towards free tubulin [69].

Overall, NCAM2 is essential in the process of neuronal differentiation. Its distribution throughout the neuron and the interactions with proteins that regulate the dynamics of the cytoskeleton enable it to participate in the development of both the dendritic tree and the axonal terminal. NCAM2 is crucial for the microtubule stabilization and the proper neuronal cytoskeleton organization; both processes are required for the maturation of neurites that became dendrites and axons. 

#### 3.2.3. NCAM2 in Synaptogenesis and Synaptic Plasticity

During brain development, neuronal polarization and synaptogenesis occur in parallel. The process of stabilization of synaptic contacts is dependent on cytoskeleton rearrangement and neuronal activity. Synaptogenesis is highly regulated by cell adhesion molecules, which control the recognition of membranes, allow the formation of synaptic structures and modulate the neuronal maturation through calcium signaling [132,133,134,135].

**Figure 3:**
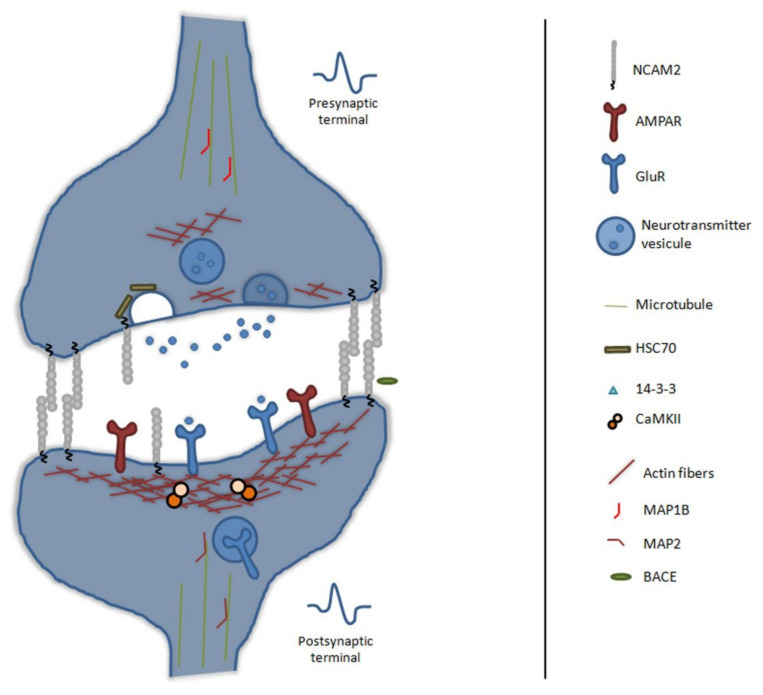
Schematic representation of NCAM2 functions and interactions in synapse maintenance and plasticity. NCAM2 is involved in synapses formation, it is detected in the presynaptic and postsynaptic compartments and undergoes *trans*-homophilic binding between both compartments. NCAM2 interacts with different scaffold proteins or complexes, which control the shape and dynamics of synapses; such as CaMKII, Actin, 14-3-3 or CAPZ. NCAM2 is involved in synaptic transmission through the neurotransmitters vesicles recycling pathway and the amount of glutamate receptors. Proteolytic cleavage of NCAM2 by BACE1 is important for synapse plasticity and remodeling. Beta-site APP cleaving enzyme 1, BACE1; Microtubule associated protein 1B, MAP1B; Microtubule associated protein 2, MAP2; Calcium/calmodulin-dependent protein kinase type II, CaMKII; F-actin-capping protein, CAPZ; α-amino-3-hydroxy-5-methyl-4-isoxazolepropionic acid receptor, AMPAR; glutamate receptor, GluR and Heat shock cognate 71 kDa, HSC70.

In vivo, NCAM2 is detected in presynaptic and postsynaptic compartments and presents trans-homophilic binding between both compartments [68]—see Figure 3. During synapse formation, NCAM2 could participate in the molecular recognition of presynaptic and postsynaptic compartments, facilitate the rearrangement of the cytoskeleton through direct or indirect interaction and modulate calcium flow that facilitates neuronal stabilization. In developing cultured cortical neurons, changes in the expression of NCAM2 can cause abnormalities in synapse formation and function. The activation of NCAM2 increases calcium levels via activation of L-type voltage-dependent calcium channels (VDCCs); these channels are highly expressed in synapses and along the dendrites and axons of mature neurons, and they play a role in regulating dendritic spine morphology [128,131,132,136]. An increase in the frequency of propagating submembrane calcium spikes in neurons with elevated levels of NCAM2 results in a reduction in dendritic spine stability and in a reduced number of mature synapses [137]. Moreover, Ca^2+^ levels via VDCCs control CaMKII complex, a key protein in the process of synapse formation and plasticity. CaMKII interacts with actin filaments [138,139]; its activation due to the increase of the intracellular calcium causes the disruption of actin filaments and the remodeling of the spine structure through multiple mechanisms, such as the translocation of α-amino-3-hydroxy-5-methyl-4-isoxazolepropionic acid, AMPA, receptors to the membrane, the activation of different members of the small GTPases family and the effect on different signaling pathways [140].

In the adult brain, synaptic plasticity is crucial for learning processes and memory tasks, and it causes a restructuration of presynaptic and postsynaptic compartments. In these processes, cell adhesion molecules interact with neurotransmitter receptors and transduce the synaptic inputs to the cytoskeleton structure. NCAM2 is located at the excitatory synapses and is important for glutamate receptor positioning [76]. The disruption of NCAM2 functions at the cell surface results in the disassembly of glutamatergic synapses and reduces the amount of actin filaments in the spine [76]. Dendritic spines have an actin cytoskeleton core, which plays a key role in maintaining the shape and plasticity of the spine [141,142]. NCAM2 interacts with actin, CAPZ complex and 14-3-3. It is known that CAPZ stabilizes actin fibers [143], is localized in the postsynaptic density [144], and neuronal activity causes its clustering in the spines, which facilitates the remodeling of the structure [145]. The loss of a subunit of the CAPZ complex is associated with alterations in the formation of spines and defects in the specification of presynaptic and postsynaptic compartments, all of which leads to errors in neurotransmission [146]. 14-3-3 is a protein involved in the regulation of cofilin phosphorylation and stabilizes the actin filaments. These functions are related to the phenotype of 14-3-3-deficient animals: a reduction in dendritic tree complexity and number of spines, along with multiple behavioral problems linked to susceptibility to schizophrenia [147,148,149]. The microtubule cytoskeleton is also present in the structure of the spine. Changes in calcium levels produce a cascade of events that modify microtubule cytoskeleton. This results in the entry of microtubules into the spine and a remodeling and stabilization of the structure [150]. In this way, all proteins that interact with the actin cytoskeleton and the microtubule cytoskeleton inside the dendritic spine are crucial for its stabilization [59,98,99], such as MAP2 or MAP1B. These MAPs are associated with maintaining the number and shape of spines [151,152,153]. A reduction in either of these proteins causes spine defects and loss of synaptic transmission [120,121]. So, the function of NCAM2 in maintenance and remodeling of the spine structure would occur through the calcium influx and cytoskeleton, where NCAM2 would interact, directly or indirectly, with proteins associated with actin cytoskeleton dynamics (such as CAPZ, cofilin, 14-3-3 or CaMKII) or with the microtubule cytoskeleton (such as MAP1 or MAP2). 

All mechanisms that are involved in the release of synaptic vesicles are relevant for proper synaptic functioning. NCAM2 is detected in presynaptic compartment and interacts with Heat shock cognate 71 kDa, HSC70, and Nogo proteins [70]. On the one hand, the HSC70 protein is a key chaperone in vesicle recycling in presynaptic structures [154]. Aberrant functioning of HSC70 is associated with neurodegenerative diseases, as HSC70 prevents the aggregation of poorly folded proteins [155]. At the functional level, CHL1 is an adhesion protein of the immunoglobulin superfamily that interacts with HSC70 [156]. Through this interaction, CHL1 also controls the vesicle recycling process, and its deficiency produces errors in vesicle recycling. In a similar way to CHL1, NCAM2 could also be involved in vesicle recycling through its interaction with HSC70. On the other hand, Nogo is a protein detected in presynaptic and postsynaptic membranes [157] that controls the number of synapses, modulates plasticity processes [158,159] and is involved in axonal growth and post-traumatic plasticity processes [160]. In addition, Nogo, MAG and other proteins are structural components of myelin present in axonal tracts [70]. The detection of NCAM2 in axonal tracts supports an interaction of NCAM2 with myelin-associated proteins, such as Nogo. Overall, these data would indicate a function of NCAM2 in the presynaptic membrane, affecting vesicle recycling, and an involvement in the functioning of the presynaptic terminal. 

Proteolytic cleavage of adhesion molecules is another mechanism for remodeling synapses. In particular, different metalloproteases cleave adhesion molecules in order to remove the physical bond between presynaptic and postsynaptic membranes and thus reshape synapses. Cleavage has been described in several proteins of the immunoglobulin superfamily, such as NCAM1, L1CAM and NCAM2 [75,161]. In vivo, NCAM2 is cleaved by ADAM10 and BACE-1 and could participate in synapses plasticity and remodeling. The amount of NCAM2 in the membrane could regulate the potentiation or depression in these plasticity processes. In detail, it has been noted that the amount of adhesion molecules present in the presynaptic and postsynaptic membranes, such as neurexins and neuroligins [162] or cadherins [163], regulate neuronal plasticity. 

In summary, NCAM2 participates in the maintenance of presynaptic and postsynaptic compartments. This function explains the high expression of NCAM2 in cortex and hippocampus during late postnatal and adult stages. The trans-homophilic binding of NCAM2 between the membranes of presynaptic and postsynaptic compartments controls the structure of the synapse. Changes in the structure of these compartments are regulated by intracellular scaffolds, which are controlled by both the calcium influx and NCAM2 interactions with the cytoskeleton, directly or indirectly by cytoskeleton associated proteins. These interactions and the cleavage of NCAM2 could participate in plasticity processes by modifying the structure of synapses.

### 3.3. NCAM2 in Calcium Signaling and Homeostasis

Relevant functions of CAMs members regarding neuronal development or synaptic plasticity are vehiculated through the calcium signaling [132]. The activation of different members of CAMs induces changes in calcium concentration that are transduced by different proteins to operate in survival mechanisms, modulate cytoskeleton dynamics and activate gene transcription [132]. In the case of NCAM2, there are some studies that analyze the relation between the protein and the calcium signaling during neurite growth and synapse formation [83,137]. In detail, it has been shown that the activation of NCAM2 increases calcium concentration through Scr and VDCC channels. More studies are required to explore other mechanisms, for example, the possibility that NCAM2 increases calcium concentration through FGFR or endoplasmic reticulum (ER) as there is evidence that some NCAM2 partners also interact with the ER. Moreover, the interaction of NCAM2 with FGFR has been previously observed [132] and it is known that the interaction of different CAMs with FGFR could increase intracellular calcium concentration, promoting neurite outgrowth and dendritic development. 

## 4. NCAM2 in Neuronal Diseases

NCAM2 is expressed during brain development as well as in adulthood and its functions are crucial for the proper cognitive process. Genetic and molecular studies show that changes in NCAM2 expression could be the cause of different pathologies both during brain development and in the adult stages—see Table 3.

### 4.1. Neurodevelopment Diseases

NCAM2 is necessary both for the correct neuronal migration and for the appropriate development of dendritic trees and axonal compartments in cortical neurons and olfactory bulb neurons. Upon NCAM2 knockdown, cortical neurons lacked a clear main apical dendrite and showed numerous primary dendrites arising from any position in the cell body [69]. This phenotype could explain the implication of NCAM2 in neurodevelopment disorders. Alterations in NCAM2 have been associated with different disorders, such as Down Syndrome or Autism Spectrum Disorders [23,67,164,165,166]. Due to its location in human chromosome 21, NCAM2 has been proposed as a candidate for the intellectual disability phenotype observed in Down Syndrome [67,166]. Even though *Ncam2* is located outside the critical region of chromosome 21, it is believed that an increased expression of NCAM2 could negatively affect the nervous system development due to dosage-related effects. In addition, genetic analyses revealed single nucleotide polymorphisms in NCAM2 gene in patients with abnormal neurodevelopment [167], Marden–Walker syndrome patients [170] and Autism Spectrum Disorders [23,164,165]. Not only does NCAM2 regulate neuronal polarization and formation of axo-dendritic compartments, but it also interacts with different cytoskeleton components that are linked to autism disorder [69,70,171,172,173]. Genetic variations in the NCAM2 gene are less known but could be the cause of intellectual disability. Therefore, one hypothesis is that structural alterations observed in patients with aberrant neurodevelopment and autism could be triggered by cytoskeleton dynamics modifications produced by variations in NCAM2 gene expression.

### 4.2. Neurodegenerative Diseases

Changes in the density and structure of the spines have been associated with the early stages of neurodegenerative diseases. At a molecular level, changes in the expression and cellular localization of adhesion proteins have been described in these diseases. Genetic studies established a relation between NCAM2 and Alzheimer’s Disease [168,169]. It has been proposed that the early loss of synapses detected in patients with Alzheimer’s Disease could be the result of the β-amyloid-induced proteolysis of synaptic NCAM2. Due to the pivotal role of NCAM2 in synaptic structures and the mentioned interactions of NCAM2 with the cytoskeleton, the ablation of NCAM2 in synapses could lead to major changes in cytoskeleton structures, thus compromising synaptic viability in Alzheimer’s Disease [76,174,175,176,177]. The interaction of NCAM2 with Granulin, GRN, one of the principal proteins associated with familial frontotemporal dementia, suggest a plausible role of NCAM2 as a receptor of the protein and a possible implication in frontotemporal dementia [70,178]. A decrease in NCAM2 levels and a change in NCAM2 localization in the spine could be associated with loss of structure of synapses in the early stages of neurodegenerative diseases.

## 5. Conclusions

**Figure 4:**
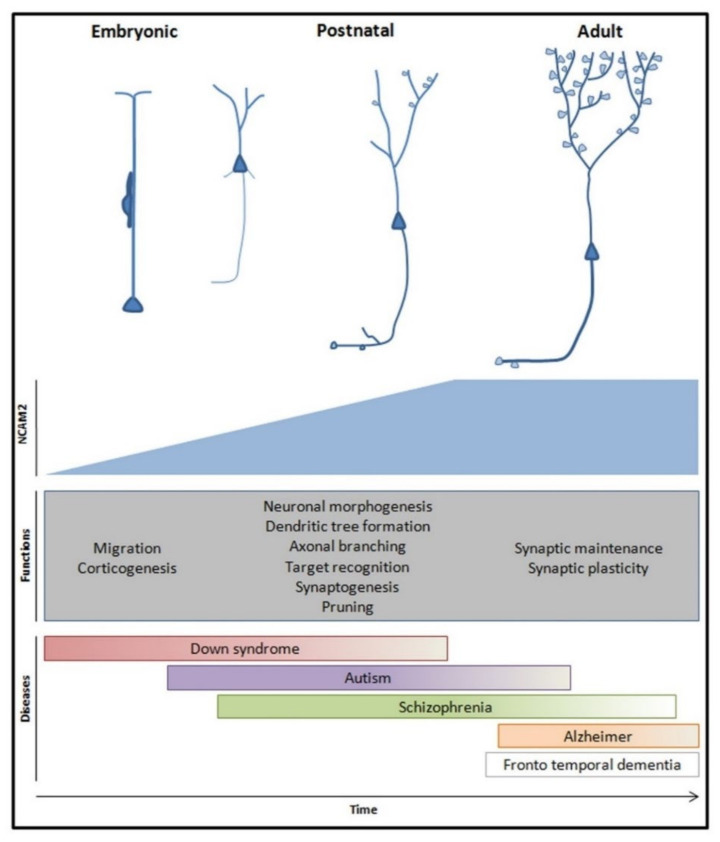
Global representation of NCAM2 physiological functions and pathological implications during brain development and in adult stages. NCAM2 expression increases during embryonic and early postnatal stages and it is maintained in adulthood. NCAM2 participates in the establishment of the neuronal molecular identity which is essential for an amount of processes during neuron development and neuronal network morphogenesis; e.g., neuronal migration, dendritic tree development, synaptogenesis and synaptic plasticity. These functions and genetic studies of neuronal pathologies showed that NCAM2 could be an important target for new therapeutic strategies.

With all the data and observations presented in this review, we can hypothesize that NCAM2 contributes to the neuronal molecular identity and is crucial for the neuronal functions—see Figure 4. In the development of the nervous system, NCAM2 regulates the molecular recognition that allows the formation of contacts between axonal and dendritic compartments leading to the formation of synapses and neural circuits. In particular, NCAM2 is present in both axonal and dendritic compartments and establishes homophilic junctions and intracellular interactions. These interactions regulate the modification of the cytoskeleton and affect the structure of the dendrite and axon. Different mechanisms are modulated by NCAM2 interactions but these can be divided in two groups: calcium signaling mechanisms and structuring of the cytoskeleton. NCAM2 is essential for the proper development of dendritic and axonal structures; its depletion causes aberrant neuron migration and differentiation and is detected in neurodevelopmental diseases. With these results, we can affirm that changes in NCAM2 alter the neuronal molecular identity, produces alterations in neuronal development and conduces to an aberrant neuronal network formation. Moreover, different CAMs modulate the neuronal differentiation, structure pruning and synaptic stabilization through the activation of gene expression. It is known that calcium influx and CaMKII modulate gene expression. More studies are necessary to identify the possible role of NCAM2 in gene expression.

In the adult brain, the neuronal network is less plastic, the dendrites and axons are stable structures and neuronal plasticity is restricted to synapses and adult neurogenesis. For synaptic plasticity to take place, neuronal molecular identity and a proper balance between stabilization of membranes and remodeling of contacts are necessary. NCAM2 is present in membranes in both synaptic compartments: presynaptic and postsynaptic. NCAM2 participates in plasticity processes through the diverse interactions in the membrane and with both presynaptic and postsynaptic scaffold structures. The amount of NCAM2 in the membrane could regulate the synaptic plasticity processes. NCAM2 controls the cytoskeleton structure and dynamics, which proves crucial for the maintenance of the synaptic scaffold and neuronal synapses. Moreover, NCAM2 could participate in local transcription, as it interacts with different transcription complexes. NCAM2 would be controlling the specific transcription of proteins in synapses, a necessary mechanism for synapse remodeling and potentiation. Besides, NCAM2 cleavage by metalloproteinases is another mechanism for synapse remodeling. Some processes controlled by NCAM2 are altered at the early stages of neurodegenerative diseases, such as Alzheimer’s disease. So, NCAM2 could represent a therapeutic target for a new strategy to preserve the synapse structure.

## Figures and Tables

**Table 1:** Involvement of Cell Adhesion Molecules in disease.

Autism Spectrum Disorder			
Molecule	Type of Study	Implications	References
CDHs	Genetic in humans	CNVs and SNPs in CDHs genes found in ASD patients.	[19,20]
PDCH	Genetic in humans	Alterations in PCDH9, PCDH10 and PCDH19 genes in patients with autism.	[21,22]
FAT1	Genetic in humans	Genetic modifications in FAT1 in Autism Spectrum Disorder patients.	[23,24,25,26]
NRXN	Genetic in humans	Mutations and CNVs in NRXN1-3 genes are associated with ASD.	[27,28]
	Experimental in mouse	NRXN1 deletion causes electrophysiological and behavioral changes consistent with cognitive impairments.	[29]
NLGN	Genetic in humans	Genetic modifications in NLGN1-4 genes found in ASD patients.	[30,31]
	Experimental in mice models	*Ngln3* and *Ngln4* KO mice displayed reduced social interaction	[32]
CNTNAP2	Genetic in humans	Genetic alterations in ASD patients	[33,34]
	Experimental in mice models	CNTNAP2 deficient mice present deficits in communication and social interaction; and repetitive behaviors.	[35,36,37]
CNTN	Genetic in humans	CNTN3–6 are considered as gene risk for ASD.	[21,30]
**Schizophrenia**			
**Molecule**	**Type of Study**	**Implications**	**References**
NCAM1	Genetic in humans	SNPs in *NCAM1* in schizophrenia and bipolar patients.	[38,39]
NLGN1	GWAS study	NLGN1 contributed to schizophrenia susceptibility in Han Chinese population.	[17,40]
Selectin	Proteomic in humans	Reduced levels in plasma from adolescents with early-onset psycosis.	[41]
VCAM1	Proteomic in humans	Reduced levels in plasma from adolescents with early-onset psycosis.	[41]
**Epilepsy**			
**Molecule**	**Type of Study**	**Implications**	**References**
N-cadherin	Mouse model	N-cadherin reduction changes mature excitatory and inhibitory circuits and contribute to significant impairment in spatial memory	[42]
	Mouse model	N-cadherin antibody alleviate brain pathology	[43]
DSCAML1	Genetic in humans	single nucleotide substitution resulting in its loss of function of DSCAML1.	[44,45]
	Rat model	GABAergic neurons were reduced and neurons’ excitability was enhanced.	[46]
NCAM1	Proteomics in CSF	NCAM1 concentration in CSF is lower in epilepsy patients.	[47]
β-integrin	Mouse model	Seizure activity and nervous system hyperexcitability.	[48]
**Fragile X Syndrome**			
**Molecule**	**Type of Study**	**Implications**	**References**
DSCAML1	Mouse model	Dosage variations of DSCAML1 disrupt cell–cell and cell–environment interactions crucial for neuronal migration and brain formation	[49]
NLGNs	Mouse model	FMRP controls the synaptic level of NLGNs	[50]
**Down Syndrome**			
**Molecule**	**Type of Study**	**Implications**	**References**
DSCAML1	Mouse model	Overexpression of DSCAM led to the inhibition of dendritic branching, a phenotype observed in DS patients.	[51]
	IPSC from DS patients	DSCAM/PAK1 pathway suppression reverses neurogenesis deficits.	[52]

**Table 2:** NCAM2 interactions and post-translational modifications.

Extracellular Region	
Interaction	References
Fibroblast Growth Factor Receptor (FGFR)	[80]
Epidermal Growth Factor Receptor (EGFR)	[79]
Nogo	[70]
Granulin	[70]
Prion protein (Prp)	[81,82]
Beta-site APP cleaving enzyme 1 (BACE1)	[75]
**Post-translational modification**	
N-glycosylation	[78]
Proteolytic cleavage	[75,76]
**Intracellular region**	
**Interaction**	**References**
Proto-oncogene tyrosine-protein kinase Src	[83]
Calcium/calmodulin-dependent protein kinase type II (CaMKII)	[69,83]
Microtubule-associated protein 2 (MAP2)	[69]
Actin	[70]
Tubulin	[69]
14-3-3 family proteins	[69,81,82]
Microtubule-associated protein 1B (MAP1B)	[70]
F-actin-capping complex (CAPZ)	[70]
Heat shock cognate 71 protein (HSC70)	[70]
**Postranslational modification**	
Palmitoylation	[70,74]
Phosphorylation	[73]

**Table 3:** NCAM2 implications in neurodevelopmental disorders and neurodegenerative diseases.

Neurodevelopmental Disorder			
Disorder	Type of Study	Implications	References
Autism Spectrum Disorders	Genetic in humans	Genetic studies associate alterations and deletions in *Ncam2* with ASD.	[23,164,165]
Down Syndrome	Genetic in humans	Increased expression in DS patients due to the location of *Ncam2* in the 21 chromosome.	[67,166]
Other neurodevelopmental disorders	Genetic in humans	Deletions *Ncam2* are found in patients with neurodevelpmental disorders.	[167]
**Neurodegenerative diseases**			
**Disorder**		**Implications**	**References**
Alzheimer’s Disease	Genetic in humans	Alterations in *Ncam2* found in AD patients.	[168,169]
	Experimental with human and mouse samples.	β-amyloid induces proteolysis of synaptic NCAM2.	[76]
Frontotemporal dementia	Experimental with mouse tissue samples	NCAM2 proposed as a candidate receptor for GRN	[70]
